# The maize ZmCPK39-ZmKnox2 module regulates plant height

**DOI:** 10.1007/s42994-024-00150-y

**Published:** 2024-03-15

**Authors:** Mang Zhu, Chenyu Guo, Xiaohui Zhang, Yulin Liu, Xiaohui Jiang, Limei Chen, Mingliang Xu

**Affiliations:** 1 State Key Laboratory of Plant Environmental Resilience, China Agricultural University, Beijing, 100193 China; 2 National Maize Improvement Center, College of Agronomy and Biotechnology, China Agricultural University, Beijing, 100193 China; 3 Center for Crop Functional Genomics and Molecular Breeding, China Agricultural University, Beijing, 100193 China

**Keywords:** Maize, Plant height, ZmCPK39, ZmKnox2, Plant hormones, Photosynthesis pathway

## Abstract

Plant height is an important agronomic trait that affects high-density tolerance and lodging resistance. However, the regulators and their underlying molecular mechanisms controlling plant height in maize remain understudied. Here, we report that knockout mutants of the calcium-dependent protein kinase gene *ZmCPK39* (*ZmCPK39*-KO) exhibit dramatically reduced plant height, characterized by shorter internodes and a slight decrease in node numbers. Furthermore, we identified a ZmCPK39-interacting protein, the knotted-related homeobox (ZmKnox2), and observed that plant height was also significantly reduced in a *mutator* transposon-inserted mutant of *ZmKnox2* (*ZmKnox2*-Mu). Combined analysis of transcriptomic and metabonomic data indicates that multiple phytohormone signaling and photosynthesis pathways are disrupted in both *ZmCPK39*-KO and *ZmKnox2*-Mu mutants. Taken together, these results provide new insights into the function of *ZmCPK39* and identify potential targets for breeding lodging-resistant and high-density tolerant maize cultivars.

**Supplementary Information:**

The online version contains supplementary material available at 10.1007/s42994-024-00150-y.

Dear Editor,

Maize (*Zea mays* L.) is one of the most important crops, providing calories and biofuel for humans. Over the past decades, the dramatic increase in corn yield has benefited from increased planting densities (Mansfield and Mumm 2014). Short stature in maize enhances grain yield by increasing lodging resistance and planting density. Although dwarf and semi-dwarf breeding has been successful in rice and wheat, it has not been effectively applied to maize, due to the yield compromise caused by dwarf plants. Although many genes regulating plant height have been identified in maize, the number of cloned genes is still limited compared to the complexity of plant height regulation (Wang et al. 2023). Therefore, identifying new plant height regulators and uncovering their underlying molecular mechanisms could facilitate the breeding of high-density-tolerant and semi-dwarf maize varieties.

Homeobox proteins can be divided into two superclasses: the homeodomain leucine zipper (HD-Zip) superclass and the three-amino-acid loop extension (TALE) superclass (Mukherjee et al. 2009). The Knotted1-like homeobox (KNOX) genes, which encode homeodomain-containing transcription factors, belong to the TALE superclass and act as master regulators in plant development (Hay and Tsiantis 2010). KN1, the first cloned member of the KNOX family, functions in plant meristems (Hake et al. 1995). It is also involved in Gibberellin acid (GA) signaling, directly regulating *GA2ox1* (Bolduc and Hake 2009). The role of KNOX genes in plant development has been exhaustively reviewed (Hake et al. 2004; Hay and Tsiantis 2010).

Calcium-dependent protein kinases (CPKs) are involved in plant growth regulation, signal transduction, and responses to both abiotic and biotic stresses (Boudsocq and Sheen 2013; Schulz et al. 2013). A notable example is AtCPK28, which has been shown to regulate stem elongation and vascular development in *Arabidopsis* (Matschi et al. 2013). Remarkably, the function of AtCPK28 in development is strictly growth phase-dependent and is associated with tissue-specific alterations in Jasmonic acid (JA) levels (Matschi et al. 2015). However, the role of CPKs in regulating plant development in maize remains largely unexplored.

In this study, we conducted a phylogenic analysis of calcium-dependent protein kinases (CPKs) in maize, together with AtCPK28 in *Arabidopsis*. Among the 41 CPKs identified in the maize genome, ZmCPK39 showed the highest homology with AtCPK28 (Fig. S1). To test whether ZmCPK39 is involved in regulating plant height, we engineered three *ZmCPK39*-knockout mutants (*ZmCPK39*-KO) using CRISPR/Cas9 technology. As expected, these mutants exhibited significantly reduced plant and ear heights compared to the WT line ND101 (Fig. 1A–C). Compared to ND101, the internodes of these mutants, particularly the top four to eight internodes, were severely shortened (Fig. 1D). Additionally, two of the three mutants had a reduced number of nodes (Fig. 1E). In summary, the knockout of *ZmCPK39* causes a severe growth retardation in stem elongation.

**Fig. 1:**
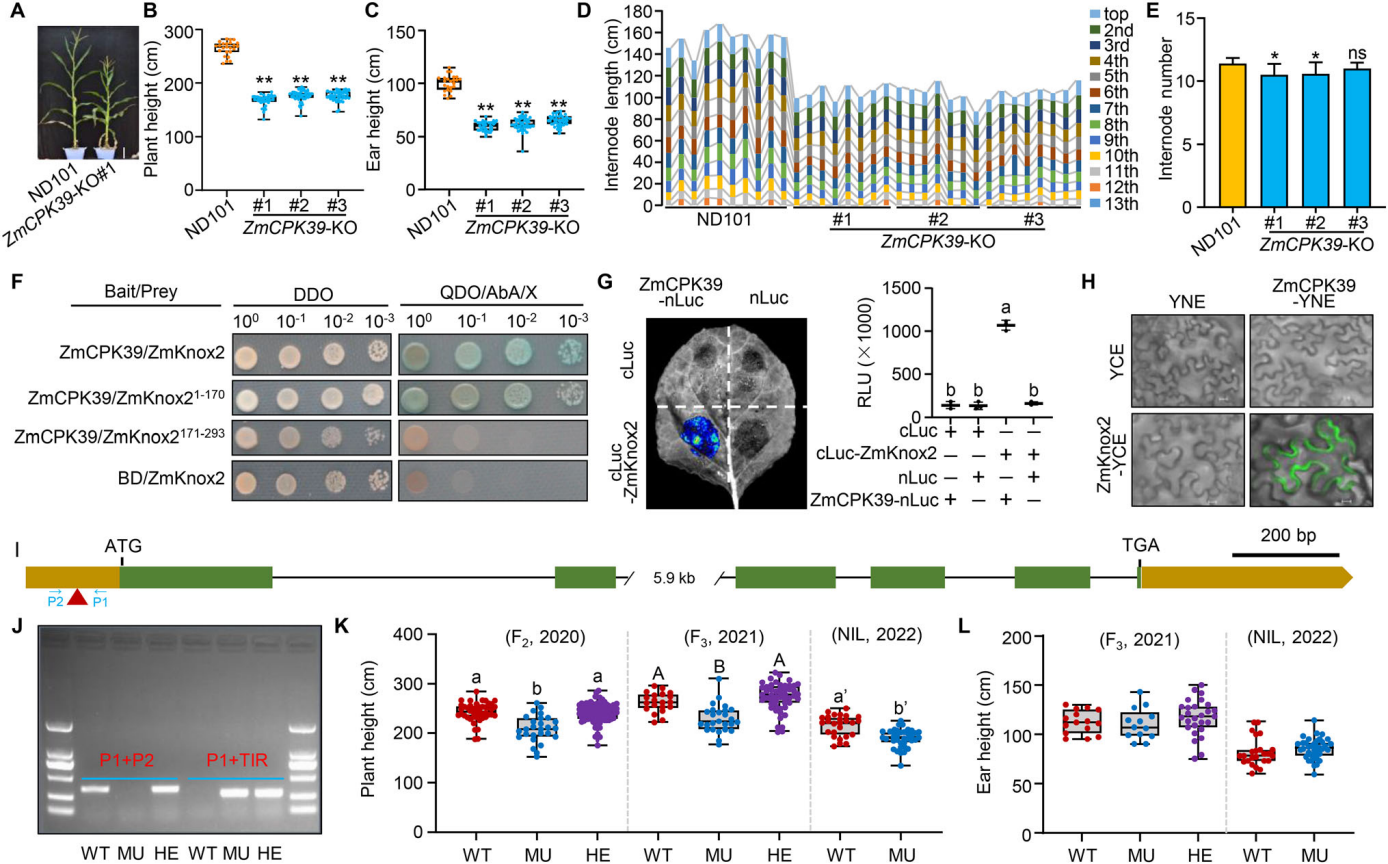
Interaction of ZmCPK39 with ZmKnox2 and their collective regulation of plant height in maize. **A** Gross morphologies of ND101 and *ZmCPK39*-KO lines. Bar, 19 cm. **B**, **C** Statistical analysis of plant height (**B**) and ear height (**C**). **D** Measurement of internode length. **E** Statistical analysis of internode number. **F** interaction of ZmCPK39 with ZmKnox2 in yeast cells. **G**, **H** Split-Luc (**G**) and BiFC (**H**) assays in *N. benthamiana* leaves to verify the interaction between ZmCPK39 and ZmKnox2. Bar, 20 μm. **I** Gene structure of *ZmKnox2* and the *Mutator* insertion site in the UTR.** J** PCR assay to verify the *Mutator* insertion. TIR, P1 and P2 are primers used to detect the *Mutator* insertion. TIR is *Mutator* specific primer, and the location of P1 and P2 are present in I. **K** *ZmKnox2*-MU showed reduced plant height. **L** *ZmKnxo2*-MU exhibited ear height comparable to WT

To elucidate the components involved in ZmCPK39-regulated plant growth, we screened for ZmCPK39-interacting proteins using a yeast two-hybrid (Y2H) assay. This screening identified a knotted-related homeobox protein, named ZmKnox2. To further verify the interaction between ZmCPK39 and ZmKnox2, we performed targeted Y2H assays in yeast. The results confirmed that ZmCPK39 indeed interacts with ZmKnox2 (Fig. 1F). Additionally, we divided ZmKnox2 into two segments: the N-terminus^1−170^ containing two KNOX domains, and the C-terminus^171−293^ comprising ELK and HOX domains. The targeted Y2H assays clearly demonstrated that ZmCPK39 interacts exclusively with the N-terminus^1−170^ of ZmKnox2 (Fig. 1F). Furthermore, the interaction between ZmCPK39 and ZmKnox2 was confirmed by split-luciferase complementation (SLC) and bimolecular fluorescence complementation (BiFC) assays (Fig. 1G, H). Additionally, this interaction between ZmCPK39 and ZmKnox2 was observed at the plasma membrane (Fig. 1H). These results demonstrated that ZmCPK39 interacts with ZmKnox2, in vivo.

To illustrate the role of *ZmKnox2* in regulating maize plant height, we acquired the *Mutator* (*Mu*) transposon-inserted line, MU2006, which is in the background of the maize inbred line W22, from MaizeGDB (https://www.maizegdb.org/). The presence of *Mu* insertion in the 5’-untranslated region (UTR) of *ZmKnox2* was confirmed by polymerase chain reaction (PCR) (Fig. 1I, J). To eliminate the effects of other *Mu* insertions or genomic variations, as well as genetic background differences between the wild-type W22 and MU2006, we crossed these lines to generate an F_2_ segregation population. In this F_2_ population, individuals homozygous for the *Mu* insertion (MU) exhibited significantly reduced plant height compared to their siblings heterozygous for the *Mu* insertion and to the plants without the *Mu* insertion (Fig. 1K). Furthermore, plants heterozygous for the *Mu* insertion were selfed to produce F_3_ families, in which plants homozygous for the *Mu* insertion at *ZmKnox2* also showed a marked reduction in plant height (Fig. 1K). Additionally, we produced a pair of near-isogenic lines (NIL) with or without homozygous *Mu* insertion. Lines with *Mu* showed lower plant height than lines without *Mu* insertion (Fig. 1K). However, plants with *Mutator*-induced *ZmKnox2* mutations exhibited ear heights comparable to those of the WT plants (Fig. 1L). These observations supported the notion that *ZmKnox2* is involved in the regulation of plant height in maize.

We conducted a transcriptomic analysis to illustrate the potential molecular mechanism underlying the regulation of plant height by the ZmCPK39-ZmKnox2 module in maize. In total, we identified 5937 down-regulated and 6792 up-regulated differentially expressed genes (DEGs) in *ZmCPK39*-KO mutants compared to the ND101 line (Fig. 2A). When comparing *ZmKnox2*-MU to WT plants, 3721 DEGs were identified, with 1882 being down-regulated and 1839 up-regulated (Fig. 2A). Surprisingly, a majority of the *KNOX* genes (8 out of 10) were down-regulated in *ZmCPK39*-KO compared to ND101 (Fig. 2B). However, in *ZmKnox2*-MU mutants, only two genes, *ZmKnox2* and *ZmKnox10-2*, were down-regulated compared to WT (Fig. 2B). In contrast, two other genes, *KN1* and *ZmKnox6*, were up-regulated in *ZmKnox2*-MU (Fig. 2B). These findings might explain why *ZmCPK39*-KO mutants exhibited a 40% reduction in plant height, whereas *ZmKnox2*-MU mutants showed only a 10% reduction.

**Fig. 2:**
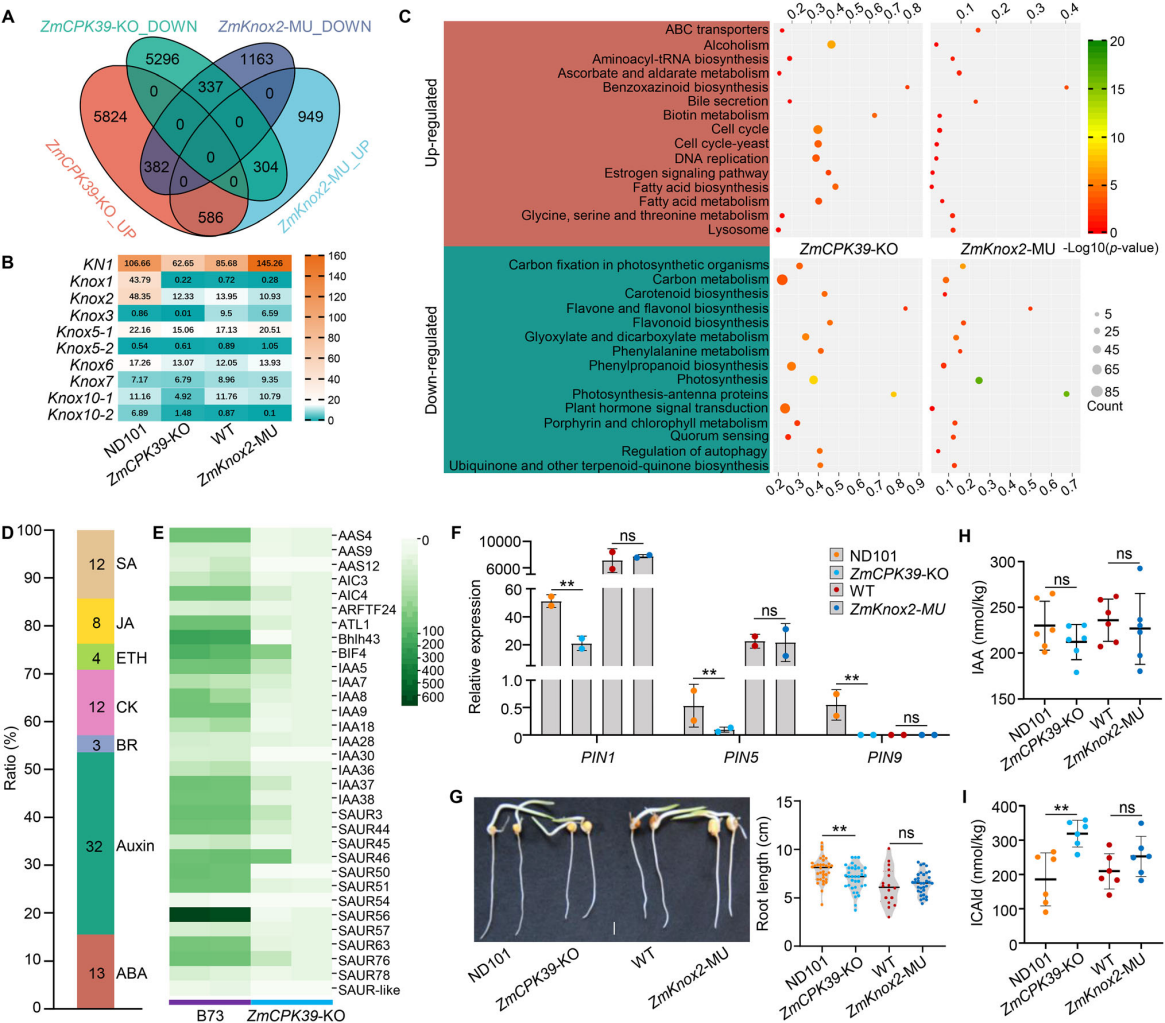
Transcriptome analysis revealed the potential mechanism underlying ZmCPK39- regulated plant height in maize. **A** Venn diagram shows DEGs in the transcriptome analysis. **B** Expression levels of the KNOTTED family members. **C** KEGG analysis for the identified DEGs. **D** DEGs involved in plant hormone signal transduction. **E** Auxin signaling-related DEGs between *ZmCPK39*-KO and ND101. **F** Expression of auxin transport genes. **G** Root length of ND101, *ZmCPK39*-KO, WT, and *ZmKnox2*-MU seedlings. Bar, 1 cm. **H**, **I** Contents of IAA (**H**) and ICAld (**I**) in ND101, *ZmCPK39*-KO, WT and *ZmKnox2*-MU. Statistical significance is indicated by asterisks: *P < 0.05, **P < 0.01, determined by *t*-test

Kyoto Encyclopedia of Genes and Genomes (KEGG) analysis revealed that the down-regulated genes in the *ZmCPK39*-KO mutants were mainly categorized into “plant hormone signal transduction”, “photosynthesis”, “phenylpropanoid biosynthesis”, and “carbon metabolism”; while up-regulated genes were involved in “alcoholism”, “fatty acid metabolism”, “DNA replication”, and “cell cycle” (Fig. 2C). Comparative analysis indicated that genes involved in “photosynthesis” and “carbon metabolism” were consistently down-regulated in both *ZmCPK39*-KO and *ZmKnox2*-MU mutants (Fig. 2C). Hence, we concluded that the ZmCPK39-ZmKnox2 module is likely regulating plant growth, via photosynthesis and carbon fixation/metabolism pathways.

We observed that DEGs related to plant hormones were largely enriched in *ZmCPK39*-KO, but not in *ZmKnox2*-MU mutants (Fig. 2C). Given the crucial role of plant hormones in controlling plant height, we focused on DEGs involved in the plant hormone signal transduction pathway. Among the 84 down-regulated plant hormone-related genes in *ZmCPK39*-KO, 32 were involved in auxin signaling, 13 in abscisic acid (ABA) signaling, 12 in cytokinin (CK) signaling, and 12 in salicylic acid (SA) signaling. Additionally, eight DEGs were related to jasmonic acid (JA), four to ethylene (ETH) signaling, and three to brassinolide (BR) signaling (Fig. 2D). These results suggest that the knockout of *ZmCPK39* disrupts multiple phytohormone pathways, particularly affecting signaling pathways for auxin, ABA, CK, and SA.

Among the 32 auxin signaling-related down-regulated DEGs in *ZmCPK39*-KO mutants, the majority were auxin/indoleacetic acid (Aux/IAAs) and small auxin-up RNA (*SAURs*) genes (Fig. 2E), known as early auxin-responsive genes (Abel and Theologis 1996). This indicates that ZmCPK39 plays a positive role in regulating auxin-responsive signaling. Notably, auxin transport genes, such as *PIN1*, *PIN5*, and *PIN9*, were down-regulated in *ZmCPK39*-KO compared to ND101, while no significant difference was observed between *ZmKnox2*-MU and WT (Fig. 2F). Correspondingly, root elongation was significantly inhibited in *ZmCPK39*-KO compared to ND101, but there was no noticeable difference between *ZmKnox2*-MU and WT (Fig. 2G). To further explore the role of ZmCPK39 in auxin signaling, we measured the auxin levels in both *ZmCPK39*-KO and ND101 lines, as well as in *ZmKnox2*-MU and WT plants. The *ZmCPK39*-KO line exhibited slightly lower, albeit not significantly different, indole-3-acetic acid (IAA) levels compared to ND101 (Fig. 2H). Additionally, the IAA-derived product indole-3-carboxaldehyde (ICAld) was significantly higher in *ZmCPK39*-KO than in ND101 (Fig. 2I). On the contrary, no difference in IAA and ICAld contents was detected between *ZmKnox2*-MU mutant and WT plants (Fig. 2H, I). Taken together, the *ZmCPK39*-KO mutant, but not the *ZmKnox2*-MU mutant, demonstrates disturbances in multiple phytohormone pathways, particularly in auxin signaling.

It is noteworthy that while *ZmCPK39*-KO showed a dramatic reduction in plant height, *ZmKnox2*-MU exhibited only a slight decrease. This disparity suggests that additional signaling pathways, such as auxin signaling, might also play a role in the regulation of plant height by ZmCPK39. Another possible explanation is the functional redundancy of other *KNOXs*, such as *KN1*, which may compensate for the *ZmKnox2*-MU. Additionally, the *Mutator* inserts into the UTR of *ZmKnox2*, rather than a complete knockout, could account for the observed effects. Considering the widespread use of hybrids in maize production, evaluating the breeding value of *ZmCPK39* and *ZmKnox2* in hybrids across various plant densities could be beneficial. Overall, our findings highlight *ZmCPK39* and *ZmKnox2* as two potential targets for the breeding of dwarf or semi-dwarf varieties using genome editing technology.

## Materials and methods

### Plant materials

The transgenic maize lines were generated using ND101 as the recipient line by the Center for Crop Functional Genomic and Molecular Breeding at China Agricultural University. A *Mutator* transposon-inserted mutant in the W22 genetic background was obtained from MaizeGDB (https://www.maizegdb.org/). This mutant was then crossed with the wild-type (WT) W22 to generate segregated populations.

### Total RNA extraction and transcription assay

Total RNA was extracted using the EasyPure Plant RNA kit (TransGen, ER301), and first-strand cDNA was synthesized using the cDNA synthesis kit (TransGen, AE311), following the manufacturer’s protocols. Quantitative Real-time PCR (qRT-PCR) was performed using the TB Green Fast qPCR mix (TaKaRa). The transcript level of *ZmGAPDH* was used as an internal control.

### Yeast two-hybrid assay

To verify the interaction between ZmCPK39 and ZmKnox2, the full-length cDNAs of *ZmCPK39* and *ZmKnox2* were cloned into the pGBKT7 and pGADT7 vectors, serving as bait and prey, respectively. The yeast strain Y2HGold was used for this assay. Both the bait and prey vectors were co-transformed into the Y2HGold strain, and the protein interactions were subsequently tested on a selective medium.

### Split luciferase complementation (SLC) assay

The full-length cDNAs of *ZmCPK39* and *ZmKnox2* were cloned into the JW771-35S-nLuc and JW772-35S-cLuc vectors, respectively, to generate the ZmCPK39-nLuc and cLuc-ZmKnox2 fusion constructs. The SLC assay was performed as previously described (Zhong et al. 2024).

## Supplementary Information

Below is the link to the electronic supplementary material.

Supplementary file1 (DOCX 41287 KB)

## Data Availability

The data are available from the corresponding author upon reasonable request.
