# Supplementary material for: The maize ZmCPK39-ZmKnox2 module regulates plant height
Source: aBIOTECH. 2024 Mar 15;5(3):356–61. doi: 10.1007/s42994-024-00150-y (PMC11399543; doi:10.1007/s42994-024-00150-y)
Supplement: Supplementary file 1 — Supplementary file1 (DOCX 41287 KB) [file 42994_2024_150_MOESM1_ESM.docx]

**Fig. S1** Phylogenic relationship between AtCPK28 and maize calcium-dependent protein kinases.
